# Marital Status, Education, and Risk of Acute Myocardial Infarction in Mainland China: The INTER-HEART Study

**DOI:** 10.2188/jea.JE20100175

**Published:** 2012-03-05

**Authors:** Bo Hu, Wei Li, Xingyu Wang, Lisheng Liu, Koon Teo, Salim Yusuf

**Affiliations:** 1 Cardiovascular Institute & Fuwai Hospital, Chinese Academy of Medical Sciences, Peking Union Medical College, Beijing, China; 2 Beijing Hypertension League Institute, Beijing, China; 3 Population Health Research Institute and McMaster University, Hamilton, ON, Canada

**Keywords:** acute myocardial infarction, marital status, level of education, case control study, coronary heart disease

## Abstract

**Background:**

We investigated the effects of marital status and education on the risk of acute myocardial infarction (AMI) in a large-scale case-control study in China.

**Methods:**

This study was part of the INTER-HEART China case-control study. The main outcome measure was first AMI. Incident cases of AMI and control patients with no past history of heart disease were recruited. Controls were matching by age (±5 years) and sex. Marital status was combined into 2 categories: single and not single. Education level was classified into 2 categories: 8 years or less and more than 8 years.

**Results:**

From 1999 to 2002, we recruited 2909 cases and 2947 controls from 17 cities. After adjustment for age, sex, BMI, psychosocial factors, lifestyle, other factors, and mutually for other risk factors, the odds ratio (OR) for AMI associated with being single was 1.51 (95% confidence interval: 1.18–1.93) overall, 1.19 (0.84–1.68; *P* = 0.072) in men and 2.00 (1.39–2.86; *P* < 0.0001) in women. The interaction of sex and marital status was statistically significant (*P* = 0.045). Compared with a high education level, a low education level increased the risk of AMI (1.45, 1.26–1.67); the odds ratios in men and women were 1.29 (1.09–1.52) and 1.55 (1.16–2.08), respectively. Single women with a low education level had a high risk of AMI (2.95, 1.99–4.37).

**Conclusions:**

Being single was consistently associated with an increased risk for AMI, particularly in women. In addition, as compared with high education level, low education level was associated with a higher risk of AMI in both men and women.

## INTRODUCTION

As compared with widowed, divorced, and single individuals, married people are more likely to engage in positive health behaviors and less likely to engage in negative health behaviors.^1^^–^^3^ Evidence Western countries suggests that marriage is associated with health benefits among men, including decreased cardiovascular morbidity and mortality.^4^ Most relevant studies have found that total and cardiovascular disease (CVD) mortality are higher among those who never married than among those who did marry.^4^^–^^9^ Before age 70 years, women have a worse prognosis than men after acute myocardial infarction (AMI).^10^^–^^12^ The causes are poorly understood. Currently the association of marriage and health among women is less consistent or of a lesser magnitude.^12^ Paradoxically, several lines of evidence suggest that marriage should be associated with better health, perhaps particularly in women.^13^ However, few studies have compared differences between sexes in the association between marital status and CVD. To our knowledge, few publications have described the relationships of marital status with all-cause and CVD mortality in a non-Western population.^14^^–^^16^ Furthermore, few studies have analyzed the effect of marital status on the occurrence of AMI in an Asian population.

Among the measures of socioeconomic status used in the present study, education is most often used, because it is easily obtained and frequently treated as a proxy for overall socioeconomic status (SES).^17^ Significant worldwide variation in the risk of AMI associated with level of education has been reported, ie, associations between AMI and education varying among regions that have different levels of socioeconomic development; however, such reports are inconsistent.^18^^–^^23^

The INTER-HEART is an international, standardized, case-control study that was designed to assess the association of AMI with several risk factors. The study measured risk factors that collectively explained over 90% of the population attributable risk (PAR) for AMI,^24^ which provided an opportunity to assess the extent to which these factors could explain the relation between marital status, level of education, and AMI.

The present study comprehensively evaluated associations between marital status, level of education, and AMI in a large-scale case-control study of a population from mainland Chinese. Interactions between sex, marital status, and level of education were also explored.

## METHODS

### Study design

This study was part of the INTER-HEART China study. INTER-HEART was a large, international, standardized, case-control study designed as a first step in assessing the importance of risk factors for acute myocardial infarction (AMI) worldwide. The objectives of INTER-HEART were to measure the strength of associations of traditional and emerging risk factors with nonfatal AMI. The 9 main risk factors were smoking, lipid levels, self-reported hypertension and diabetes, obesity, diet, physical activity, alcohol consumption, and psychosocial factors. The study design of the INTER-HEART study has been explained in detail elsewhere.^24^^,^^25^

The sample size in China was calculated on the basis of (1) a level of significance of α = 0.05 (2-sided test); (2) a power (1 − β) of 80%; (3) effect size (minimum odds ratio [OR] considered to be clinically relevant based on the risk factor of interest; for tobacco, smoking, and hypertension, ORs ≥2.0 were considered clinically significant); and (4) exposure (exposure rate in the control group was estimated on the basis of the prevalence in the general population, as shown in previous studies in China). In 2005, the Center for Research and Control of Cardiovascular Diseases reported^26^ that morbidity of AMI was 17.8% per year. The sample size was thus approximately 3000.

### Subject selection

From 1999 to 2002, we recruited 2909 cases and 2947 controls from 25 centers in 17 cities in mainland China. Study centers and cities were selected on the basis of feasibility. All patients admitted to the coronary care unit or an equivalent cardiology ward of participating centers were screened to identify incident cases of AMI. Cases were identified by using a standardized definition and enrolled within 24 hours of onset of symptoms. At least 1 age- (±5 years) and sex-matched control (without a history of CVD) was recruited per case from non-cardiac wards, unrelated visitors of cardiac patients, or patients at the same center with illnesses not obviously related to CVD or its risk factors. The inclusion criteria for cases and controls have been previously reported.^24^ At entry to the study, informed consent was obtained from each subject. The study protocol was approved by the appropriate regulatory and ethics councils in China and in all participating centers.

### Data collection

Structured and pre-test questionnaires were administered, and physical examinations were given, in the same manner for cases and controls. Data were obtained on socioeconomic status (level of education, income, occupation, and marital status), lifestyle (tobacco use, physical activity, and dietary patterns), psychological conditions, personal and family history of CVD, and risk factors (hypertension, diabetes mellitus). Psychosocial factors (depression, locus of control, perceived stress, and life events) were systematically recorded and integrated into 1 score. Standard, straightforward physical examinations were performed in duplicate by the same examiner for each subject to record anthropometric measures (weight, height, waist, and hip circumference) and heart rate. Waist-hip ratio was defined as waist circumference divided by hip circumference. Body mass index (BMI) was defined as weight (kg) divided by the square of the height (m^2^). According to Chinese guidelines for prevention and control of adult overweight and obesity, abdominal obesity was defined as a waist measurement of 90 cm or greater in men and 85 cm or greater in women. Because blood pressure in cases would be systematically affected by the myocardial infarction and treatments, only self-reported history of hypertension was used in the analysis.

Non-fasting blood samples (20 ml) were drawn from all participants and were centrifuged within 2 hours of admission, separated into 6 equal volumes, and frozen immediately at −20°C or −70°C after processing. Centers were instructed to draw blood from cases within 24 hours of symptom onset. Immunoturbidimetric assays were used to measure apolipoprotein. Because apolipoprotein concentrations are not affected by the fasting status of the individual, the ApoB/ApoA1 ratio was used as an index of abnormal lipids in the current analysis.^27^

Quality of data collection was maintained by using standardized protocols and centralized training. All data were electronically entered at each center into a customized database programmed with the appropriate ranges and then checked for consistency using quality control measures.

### Definition of variables

Marital status was classified into 6 categories: never married, currently married, common law/living with partner, widowed, separated, and divorced. Level of education was assessed using 5 categories: none, 1–8 years, 9–12 years, trade school, and college/university. Current smokers were defined as individuals who smoked any tobacco in the previous 12 month, including those who had quit during the past 1 year. Former smokers were defined as those who had quit more than 1 year earlier. Regular alcohol use was defined as alcohol consumption 3 or more times a week. Individuals were judged to be physically active if they were regularly involved in moderate exercise (walking, cycling, or gardening) or strenuous exercise (jogging, football, and vigorous swimming) for 4 or more hours a week. Vegetables and fruit consumption was defined as the number of times any vegetables or fruits were consumed in 1 week. A combined psychosocial index was devised by using a combination of parameter estimates from the fully adjusted multivariate logistic regression model.^24^ The score was based on a combination of depression vs none, stress at work/home (general stress variable) vs none, moderate/severe financial stress vs minimal/none, 1 or more life events vs none, and a locus of control score in the lower 3 quartiles of the distribution vs the top quartile. The cutoffs for ApoB/ApoA1 ratio were derived from all controls (men and women).

Taking into account the number of people in each class and the purpose of stratification, marital status was converted into 2 categories. Widowed, divorced, and separated were combined into “single”, and never married, currently married, and living with a partner/common-law spouse were combined into “not single”. In China, the duration of primary education is 8 to 9 years. To remain consistent with the International INTER-HEART study, level of education was also combined into 2 categories: low education level (≤8 years) and high education level (>8 years). All analysis was based on these categories.

### Statistical analyses

Baseline characteristics were described as mean (SD) for continuous variables and frequencies (proportions) for categorical variables. Categorical variables were compared with the chi-square test. Continuous variables were compared with *t*-test or appropriate nonparametric tests when distributional assumptions were in doubt. Multivariate logistic regression models were used to measure associations between marital status and AMI after adjusting for the 9 risk factors. The international INTER-HEART study measured several risk factors, which collectively explained over 90% of the population attributable risk (PAR) for AMI. The 9 main risk factors were smoking, lipids, self-reported hypertension and diabetes, obesity, diet, physical activity, alcohol consumption, and psychosocial factors. These risk factors were adjusted for in the logistic regression models. In addition, sex-specific associations were investigated using the logistic models. The strength and direction of the associations were indicated with odd ratios (ORs) and corresponding 95% confidence intervals (CIs). The Wald chi-square test was used to test the interaction between sex and marital status. The Mantel-Haenszel chi-square test was used as a linear trend test for ordinal variables. A *P* value of 0.05 or less was considered to indicate statistical significance. All statistical analyses were performed by using SAS 9.13 software. Figures were constructed with Graphpad Prism 4 Demo.

## RESULTS

A total of 5856 participants (2909 cases and 2947 age- and sex-matched controls) were included in the study; 541 participants—353 (12.1%) cases and 188 (6.4%) controls—were single. The proportions of single men and women were 7.0% and 24.1%, respectively, among cases and 4.5% and 10.8% among controls. A total of 2857 participants—1557 (53.5%) cases and 1300 (44.2%) controls—had a low education level. The proportions of men and women with a low education level were 42.8% and 77.6%, respectively, among cases and 36.3% and 62.0% among controls.

Table 1 shows the baseline characteristics and risk factors for AMI in all participants and by sex. Overall, low income level, history of hypertension, history of diabetes, and ApoB/ApoA1 ratio were more frequent in cases than in controls (*P* < 0.05 for all comparisons). However, vegetable and fruit intake and rates of current smoking and moderate and strenuous physical activity were lower in cases than in controls (*P* < 0.05 for all comparisons). In the subgroup analysis by sex, the distributions of factors between cases and controls were consistent with the distributions among all participants.

**Table 1.** Selected demographic characteristics of cases and controls

	Total		Men		Women	
		
	Cases (2909)	Controls (2947)	Cases (2027)	Controls (2048)	Cases (882)	Controls (899)
Age (years)	62.11 ± 11.72	60.35 ± 11.42***	59.92 ± 12.05	58.37 ± 11.72***	67.06 ± 9.05	64.60 ± 9.07***
BMI	24.68 ± 3.10	24.40 ± 2.91***	24.68 ± 2.93	24.32 ± 2.86***	24.70 ± 3.46	24.58 ± 3.02
Annual income						
<10 000 yuan	1869 (64.7%)	1782 (60.6%)**	1185 (58.7%)	1161 (56.8%)	684 (78.4%)	621 (69.4%)***
≥10 000 yuan	1021 (35.3%)	1157 (39.4%)**	833 (41.3%)	883 (43.2%)	188 (21.6%)	274 (30.6%)***
Tobacco						
Smokers	1694 (58.2%)	1186 (40.2%)***	1506 (74.6%)	1112 (54.3%)***	175 (20.0%)	73 (8.1%)***
Nonsmokers	1215 (41.8%)	1761 (59.8%)***	513 (25.4%)	935 (45.7%)***	702 (80.0%)	826 (91.9%)***
Alcohol						
Regular drinkers	1068 (37.0%)	1198 (40.8%)	988 (49.0%)	1078 (52.7%)*	80 (9.1%)	120 (13.4%)**
Nondrinkers	1824 (62.7%)	1746 (59.2%)	1028 (51.0%)	967 (47.3%)*	796 (90.9%)	779 (86.6%)**
History of hypertension						
Yes	1145 (39.4%)	656 (22.3%)***	736 (36.3%)	416 (20.3%)***	409 (46.4%)	240 (26.7%)***
No	1764 (60.6%)	2291 (77.7%)***	1291 (63.7%)	1632 (79.7%)***	473 (53.6%)	659 (73.3%)***
History of diabetes						
Yes	360 (12.4%)	87 (3.0%)***	204 (10.1%)	56 (2.7%)***	156 (17.7%)	31 (3.5%)***
No	2549 (87.6%)	2860 (97.0%)***	1832 (89.9%)	1992 (97.3%)***	726 (82.3%)	868 (96.5%)***
Psychosocial index	0.18 (0.00, 0.72)	0.18 (0.00, 0.18)***	0.18 (0.00, 0.72)	0.18 (0.00, 0.18)***	0.18 (0.00, 0.72)	0.18 (0.00, 0.18)***
Physical activity						
Moderate/strenuous	110 (3.8%)	121 (4.1%)	92 (4.5%)	98 (4.8%)	18 (2.0%)	22 (2.4%)
None/light	2799 (96.2%)	2826 (95.9%)	1935 (95.5%)	1950 (95.2%)	864 (98.0%)	877 (97.6%)
ApoB/ApoA1 ratio	0.70 ± 0.22	0.61 ± 0.20***	0.71 ± 0.21	0.62 ± 0.21***	0.71 ± 0.24	0.59 ± 0.18**
Weekly servings of ​ vegetables and fruit	19.34 ± 11.63	20.54 ± 11.32***	19.31 ± 11.69	20.41 ± 11.55**	19.42 ± 11.49	20.86 ± 10.77**

Comparison of cases and controls: *P < 0.05, **P < 0.01, ***P < 0.001. Psychosocial index is shown as median (Q1, Q3).

Single participants had an OR of 1.51 (95% CI: 1.18–1.93) for AMI as compared with those who were not single, after adjustment for all risk factors (Table 2). However, this increase in risk diminished substantially in men after adjustment for all risk factors (OR, 1.19; 95% CI, 0.84–1.68). However, the association remained significantly increased in women (2.00, 1.39–2.83) after adjustment for all risk factors. Participants with a low education level had an OR of 1.45 (1.26–1.67) for AMI as compared with those with a high education level. The odds ratios in men and women were 1.29 (1.09–1.52) and 1.55 (1.16–2.08), respectively. There was an interaction between sex and marital status (*P* = 0.045). Women who were not single had a lower rate of AMI than did men who were not single (61.0% vs 68.5%), but single women had a higher rate of AMI than did single men (49.0% vs 45.4%) (*P* < 0.05 for both comparisons).

**Table 2.** Odds ratio for AMI by marital status and education level in 4 models

	Total			Men			Women		
		
	OR	95% CI	*P*	OR	95% CI	*P*	OR	95% CI	*P*
**Marital status**									
Adjusted for age	1.89	1.56–2.30	<0.0001	1.47	1.11–1.94	<0.0001	2.25	1.71–2.96	<0.0001
Adjusted for age, tobacco	1.79	1.47–2.18	<0.0001	1.49	1.12–1.98	<0.0001	2.12	1.60–2.80	<0.0001
Adjusted for age, tobacco, hypertension, diabetes, ​ ApoB/ApoA1	1.85	1.46–2.35	<0.0001	1.48	1.06–2.07	<0.0001	2.35	1.66–3.32	<0.0001
Adjusted for all risk factors	1.51	1.18–1.93	<0.0001	1.19	0.84–1.68	0.249	2.00	1.39–2.86	<0.0001
**Education**									
Adjusted for age	1.42	1.27–1.58	<0.0001	1.25	1.10–1.43	0.0007	1.97	1.60–2.44	<0.0001
Adjusted for age, tobacco	1.33	1.19–1.49	0.0001	1.17	1.02–1.33	0.0083	1.84	1.48–2.28	<0.0001
Adjusted for age, tobacco, hypertension, diabetes, ​ ApoB/ApoA1	1.42	1.24–1.62	<0.0001	1.31	1.12–1.54	0.0002	1.70	1.31–2.22	<0.0001
Adjusted for all risk factors	1.45	1.26–1.67	<0.0001	1.29	1.09–1.52	0.0005	1.55	1.16–2.08	<0.0001

also adjusted for sex

**Figure 1.**
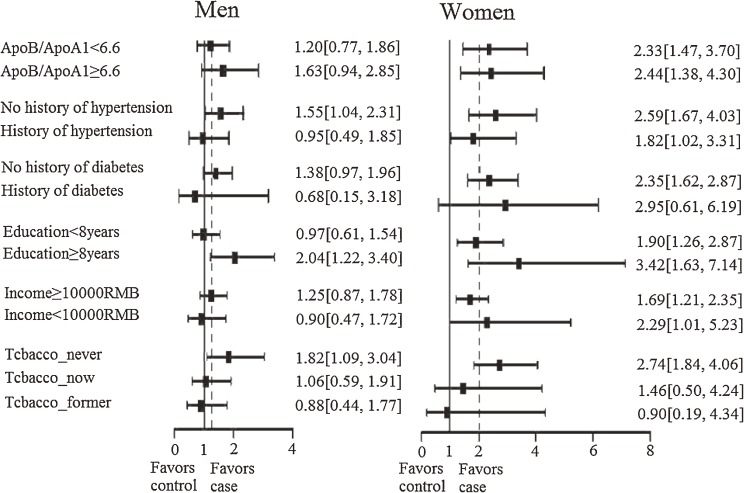
Odds ratio of myocardial infarction according to marital status, by sex and subgroup. Dotted line: odds ratio of overall were 1.19 (0.84, 1.68) in men and 2.00 (1.39, 2.86) in women. RMB: Chinese currency.

Figure 1 shows the relation between marital status and risk of AMI by sex in various subgroups. Among women, the association of marital status with risk of AMI was consistently observed in women with a high or low education level, in those with a high or low income, in those with or without history of hypertension, and in those with a high or low ApoB/ApoA1 ratio. However, among men, the association of marital status with AMI risk varied by subgroup. In most subgroups, the odds ratio for AMI associated with marital status was higher in women than in men.

**Figure 2.**
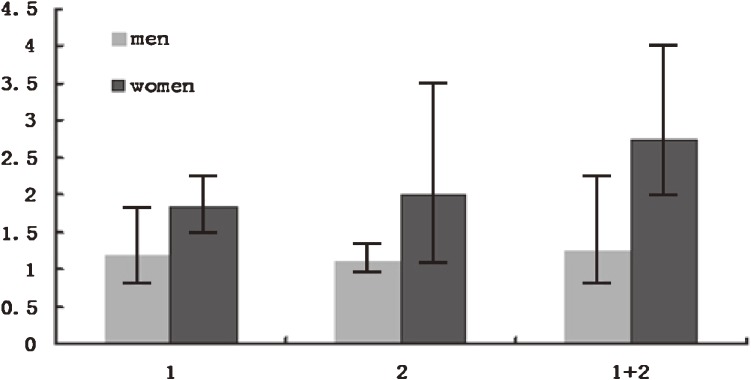
Odds ratio of AMI according to education level and marital status. (1) ≤8 years of education; (2) single; (1) + (2) both ≤8 years of education and single.

Figure 2 shows the interaction of sex, marital status, and level of education with AMI risk. Among single women with a low education level, AMI risk (OR, 2.95; 95% CI, 1.99–4.37) was concomitantly increased (*P* = 0.018 for linear trend). This trend was not present for men (*P* = 0.23).

## DISCUSSION

Being single might result in psychosocial problems and make a person feel stress at work or home. It has long been believed that stress influences long-term psychological status and subsequent development of chronic disease. Marriage generally benefits health because it provides social support and security and because married persons experience less depression and have healthier lifestyles.^2^^,^^3^ Marriage may also buffer against stress and thereby reduce activation of neuroendocrine systems,^28^ which could slow progression of atherosclerosis and other pathologic processes.^29^^–^^32^ Previous studies showed that weakened social ties and persistent social isolation were associated with increased risk of mortality from coronary heart disease (CHD)^33^ and mortality from all causes^34^ among both men and women. A Chinese epidemiologic survey showed that stress significantly increased the risk of CHD and might be an important risk factor, independent of traditional risk factors for CHD in the Chinese population.^35^

Many studies have shown that married men have a survival advantage over unmarried men.^7^^–^^9^^,^^36^ However, after adjusting for potential risk factors, the protective effect of marriage was not seen among men in the present study. However, a significant protective effect of marriage was seen in women. This disagreement among studies of the importance of marital status in men and women may be due in part to cultural differences in the populations studied. Hu and Goldman showed that the effects of being married on mortality are present in a wide variety of cultures.^37^ Reports of similar effects have emerged from the United States,^4^ Great Britain,^8^ Sweden,^38^ Denmark,^39^ and the Netherlands.^40^ In China, people are influenced by traditional feudal ideology, in which men and women were not equal in social or economic status. With economic development, this situation has improved greatly. However, many women remain in subordinate positions and are not sufficiently independent, especially in rural areas. In such environments, women are more likely to experience stress and depression and have a higher risk of AMI than do men when they become divorced, separated, or widowed. Clearly, cultural differences should be considered when comparing the disease impact of marital status in different populations.

In the present study, low education level was the marker most consistently associated with increased AMI risk. According to Spruit,^41^ education is an indicator of material and immaterial life circumstances. While acknowledging the effect of education on general values, the role of education can be extended to behavioral factors. People with more education have lower death rates than do those with less education. The inverse relationship is consistent for many cardiovascular and non-cardiovascular causes of death. Risk factors for cardiovascular disease are less frequent in those with a higher education level. Hypertension^42^ and cigarette smoking^43^ are less prevalent among those with a high education level, although serum cholesterol did not significantly differ by education level.^44^

It is worth noting that there was an interaction between living alone and low education level and increased AMI risk among women. This phenomenon was not found in men. It proves that women in China are more vulnerable than men to the impact of living alone and low education level. Because marital status and education are easily ascertained and are frequently used as proxies for socioeconomic status, these variables can be used to screen for high-risk groups, especially among women.

### Limitations

Several limitations of this study warrant consideration. An important potential disadvantage was that the present control population may not be representative of the general Chinese population and that the case patients may not be representative of all Chinese with AMI. Another potential limitation was that only surviving cases were investigated. A third limitation was that the causal relation between marital status and AMI could not be determined due to the case-control study design. Additionally, the relatively limited data collected on income and occupation may have attenuated the results of the study.

### Conclusions

The results suggest that being single increases the risk of AMI, particularly in women, and that low education level increases this risk in both women and men. Marital status and education level might thus be useful in instruments attempting to identify high-risk individuals.

## APPENDIX

### INTERHEART China Investigators

X. Chen (Baotou), G. Dong (Qingdao), Q. Fu (Xuzhou), J. Huang (Xian), R. Hui (Beijing), B. Jiang (Beijing), J. Li (Wuhan), X. Li (Nanjing), X. Li (Liaocheng), Y. Li (Zhenyang), Z. Li (Zhenyang), Y. Liao (Wuhan), X. Liu (Zhengzhou), A. Ma (Xian), J. Sanderson (Hong Kong), Z. Wan (Tianjin), D. Wang (Wuhan), F. Wang (Taian), X. Wang (Yangquan), Y. Wei (Chaoyang), T. Xu (Zuzhou), L. Zhang (Beijing), Y. Zhang (Weifang), R. Zhao (Baotou), C. Zhou (Qingdao), X. Zhu (Jinan).
